# Crystal Structure of R120G Disease Mutant of Human αB-Crystallin Domain Dimer Shows Closure of a Groove

**DOI:** 10.1016/j.jmb.2011.02.020

**Published:** 2011-04-22

**Authors:** A.R. Clark, C.E. Naylor, C. Bagnéris, N.H. Keep, C. Slingsby

**Affiliations:** Department of Biological Sciences, Crystallography, Institute of Structural and Molecular Biology, Birkbeck College, Malet Street, London WC1E 7HX, UK

**Keywords:** chaperone, disease biomarker, myopathy, proteostasis, stress response, ACD, α-crystallin domain, AP, antiparallel, Hsp, heat shock protein, sHsp, small heat shock protein, MPD, methyl pentane diol, rTEV, recombinant tobacco etch virus, His, histidine

## Abstract

Small heat shock proteins form large cytosolic assemblies from an “α-crystallin domain” (ACD) flanked by sequence extensions. Mutation of a conserved arginine in the ACD of several human small heat shock protein family members causes many common inherited diseases of the lens and neuromuscular system. The mutation R120G in αB-crystallin causes myopathy, cardiomyopathy and cataract. We have solved the X-ray structure of the excised ACD dimer of human αB R120G close to physiological pH and compared it with several recently determined wild-type vertebrate ACD dimer structures. Wild-type excised ACD dimers have a deep groove at the interface floored by a flat extended “bottom sheet.” Solid-state NMR studies of large assemblies of full-length αB-crystallin have shown that the groove is blocked in the ACD dimer by curvature of the bottom sheet. The crystal structure of R120G ACD dimer also reveals a closed groove, but here the bottom sheet is flat. Loss of Arg120 results in rearrangement of an extensive array of charged interactions across this interface. His83 and Asp80 on movable arches on either side of the interface close the groove by forming two new salt bridges. The residues involved in this extended set of ionic interactions are conserved in Hsp27, Hsp20, αA- and αB-crystallin sequences. They are not conserved in Hsp22, where mutation of the equivalent of Arg120 causes neuropathy. We speculate that the αB R120G mutation disturbs oligomer dynamics, causing the growth of large soluble oligomers that are toxic to cells by blocking essential processes.

## Graphical Abstract

**Figure:**
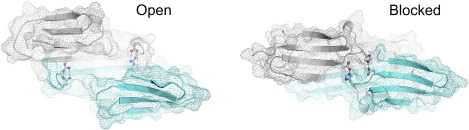

**Research Highlights:**

► Small heat shock proteins have a conserved arginine in a conserved domain (ACD). ► This arginine forms an ion pair at each end of an open groove in the normal ACD dimer. ► Mutation of this arginine in several proteins causes myopathy, neuropathy or cataract. ► Structure of R120G of αB-crystallin shows the groove is blocked by new ion pairs. ► This mutation leads to aggregation of αB-crystallin, resulting in disease.

## Introduction

All cells can protect themselves from hostile environmental changes by the stress response system, which includes increasing the levels of heat shock proteins (Hsps).^1,2^ Hsps contribute to proteostasis^3^ by binding nonnative proteins, and they are integrated into cellular networks that protect the cell from accumulating damaged proteins.^4^ Small Hsps (sHsps) form large oligomeric assemblies with a high capacity for holding onto nonnative proteins^5^ that are handed over to the ATP-driven chaperone machines for refolding.^6^ sHsps are ubiquitous among the kingdoms of life and within mammalian genomes occur in large families with members differentially expressed in many tissues including muscle, brain and the eye lens.^7–9^ Members of the family of 10 human sHsps (HSPB1–B10) are mainly cytoplasmic.^10^ αB-Crystallin (HSPB5), Hsp27 (HSPB1) and Hsp22 (HSPB8) are cytoprotective, with roles in apoptosis and autophagy.^11,12^ A cytoprotective function is a hindrance to health when exploited by cancer cells.^13^ αB-Crystallin behaves as an oncoprotein when expressed in basal breast cancer^14^ and blocks apoptosis in highly migratory glioma cells,^15^ making it a potential drug target.

αB-Crystallin is constitutively and highly expressed in the eye lens^16^ and to a lesser extent in skeletal and heart muscle.^7^ Cells in these tissues are long-lived and highly elongated, making them vulnerable to protein aggregation and deficiencies in waste-disposal processes.^17^ Either event will likely cause upregulation of stress proteins, making sHsps, in particular, biomarkers of disease in these tissues.^11,18,19^ In the brain, αB-crystallin is upregulated in neuron-supporting glial cells during stress, for example, in astrocytes subject to blockade of the proteasome.^20^ In the very early stages of multiple sclerosis, αB-crystallin is upregulated in oligodendrocytes that are adjacent to activated HLA-DR-expressing microglia.^21^ Severe challenges to proteostasis can occur on expression of stress proteins bearing dominant missense mutations as they signal further stress, leading to more dysfunctional protein. Unsurprisingly, sHsp stress proteins are associated with swathes of inherited human diseases, including cataract,^22^ skeletal myopathies,^23^ cardiomyopathies^24^ and neuropathies.^25,26^

Small Hsp sequences have in common an “α-crystallin domain” (ACD) of around 90 amino acids, with most having a short conserved C-terminal extension bearing an I-X-I motif and a longer variable N-terminal extension.^27^ Kingdom-wide sequence comparisons show subgroups of sHsp sequences between metazoans and nonmetazoans.^28,29^ Attention has focussed on an arginine within the ACD that is conserved across all phyla, as its mutation in different sHSP family members causes several human diseases. The mutation R120G in αB-crystallin causes desmin-related myopathy.^30^ The equivalent arginine mutation (R116C) in αA-crystallin (HSPB4) causes zonular central nuclear cataracts.^31^ In Hsp27 and Hsp22, the equivalent substitution (R127W and K141N/E, respectively) results in neuromuscular disorders, axonal Charcot–Marie–Tooth disease type 2F and 2L, and distal hereditary motor neuropathy.^26,32^ The sequence conservation of this arginine and the associated pathogenic mutations in the different sHsp sequences highlight that it is performing a general role common to the ACD in many sHsps.

A striking feature of sHsps is their association into large, soluble, globular assemblies with a quaternary organisation that allows a variable number of protomers.^5^ *In vitro*, recombinant αB R120G mutant protein forms soluble oligomers that are larger and more polydisperse than wild type^33^ and that are prone to aggregation and precipitation on handling.^34^ Although solution polydispersity in α-crystallins is advantageous for maintaining eye lens transparency, it is a hindrance for three-dimensional (3D) atomic characterisation.^35^ X-ray crystallography has revealed high-resolution structures of two well-defined, and hence atypical, assemblies: a 24-mer from an archaeal thermophile^36^ and a 12-mer from wheat.^37^ The ACD is an immunoglobulin-like beta sandwich comprising strands β2–β9, and the assembly unit is a dimer. The I-X-I motifs contribute to building dimers into an assembly by docking into pockets on one side of a neighbouring ACD beta sandwich. The structural role of the N-terminal extensions, which emerge from the other side of the beta sandwich, is less clearly defined. Single-particle electron microscopy studies of shell-like 24-mers from archaeal HSP16.5 indicate a buried location,^38^ whereas in 24-mers from yeast HSP26, 12 N-terminal domains are buried inside the assembly and 12 are on the surface.^39^ Only 6 N-terminal extensions were seen buried within the double-disc 12-mer in the crystal structure of wheat HSP16.9,^37^ where they contributed to holding the assembly together.

**Fig. 1:**
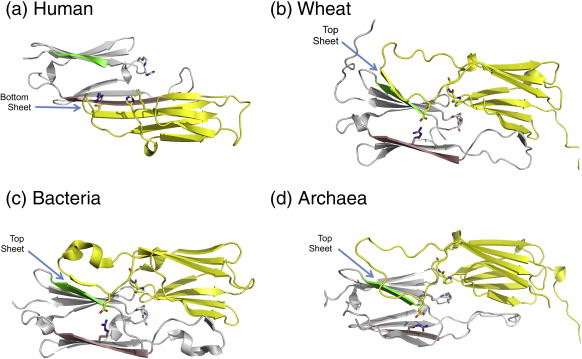
The conserved arginine forms an interchain ion pair in the ACD dimers. (a) The mammalian ACD dimer from human (2WJ7). (b) The plant ACD dimer from wheat, *Triticum aestivum* (1GME). (c) The bacterial ACD dimer, from *Xanthomonas axonopodis* (3GLA). (d) The archaeal dimer, from *Methancoccus jannaschii* (1SHS). In all structures in one chain the β7 strand (with the conserved arginine) is coloured brown, the N-terminal β2 strand is green, and the second molecule in the dimer is yellow. Also, in the β3/4 arch, the ProGly (of nonmetazoans) and LysHis in the human dimer are shown as sticks. The β-sheet ACD dimer interface that is formed by either the top or bottom β-sheet is indicated by a blue arrow.

Recent progress in crystallographic structure determination of vertebrate sHsps has relied on truncation of the flanking sequence extensions. The ACD dimers show a mode of dimerisation that is different from that of nonmetazoans (Fig 1):^40–42^ the fewer residues between strands β5 and β7 in metazoan sequences result in the loss of a distinct β6 strand, and instead, β7 strand is extended and referred to as β6 + 7 strand. This extended strand interacts about the dimer interface with the equivalent strand in the other chain, whereas in nonmetazoans the β6 strand exchanges between partner chains at the dimer interface. In effect, an interface region on one side of the ACD sandwich has switched from one sheet to the other (Fig. 1). Sequence differences support these distinct modes of dimerisation, in particular the presence of a PG motif in nonmetazoans compared with a KH motif in metazoans (Table 1). The conserved arginine in human sHsps is on the β6 + 7 strand at the dimer interface, not far from the dyad axis.

The different dimeric arrangements mean that the ACD side pockets, which are docking stations for I-X-I C-terminal motifs, are in different relative orientations in nonmetazoan and metazoan sHsps. When the I-X-I motif is included in the human αB-crystallin construct, it binds into ACD pockets, forming runaway assemblies in the crystal lattice.^41^ Recent solid-state NMR studies on full-length human αB-crystallin show the ACD dimer structure within the context of the assembled oligomer and also show the binding of the I-X-I motif into ACD pockets.^44^ The crystal structure of bovine αA-crystallin shows that the I-X-I C-terminal motif can bind in the reverse direction by exploiting its palindromic sequence.^41^ In zebrafish αA-crystallin, the motif can bind into ACD pockets formed from the same chain.^42^ These pockets are candidate-binding sites for nonnative protein substrates.^45,46^ It has been proposed that a similar motif sequence in Bag3, a co-chaperone that plays a role in macroautophagy,^47^ binds into ACD pockets of Hsp22.^12^

Although the conserved arginine occurs in two kinds of structural environment, due to the different ways in which ACDs dimerise among the kingdoms of life (Fig. 1), it does form an interchain ion pair with a topologically equivalent aspartate (mammals) or glutamate (Table 1) in sHsps of all other phyla for which high-resolution structural data are currently available. Within vertebrates, there is a source of oligomer heterogeneity that impacts on the environment of the conserved arginine and that is the register shift at the dimer interface. All the structures of the ACDs from vertebrates solved to date form an antiparallel (AP) dimer about the β6 + 7 strand (Fig. 1) that has been termed the AP interface.^41^ The register of the strands can switch, with three different registers observed (see Supplementary Fig. S1). In both AP2 and AP3 registers, the conserved interchain ion pair (Arg120–Asp109 in αB) involving the conserved arginine is maintained.

Here we have solved the X-ray structure of the ACD dimer of human αB R120G close to physiological pH, as well as higher-resolution αB ACD wild-type controls. We compared the mutant dimer structure with several recently determined wild-type vertebrate ACD dimer crystal structures and with the solid-state dimer structure resolved in the context of the full-length sequence in a large assembly in order to assign the conformational changes attributable to the R120G mutation.

## Results

### Structure of the mutant αB-crystallin ACD dimer

Two mutations improved the diffraction resolution of crystals of human αB ACD: L137M substitution and truncation of the β2-strand. The highest-resolution structure was solved from a β2-strand truncated construct, αB ACD L137SeM (residues 71–157). The structure of the human αB ACD L137SeM (residues 67–157, the same residues as in 2WJ7) was also solved. The mutation L137M is located at the surface of a β4/β8 side pocket, and in human αB ACD L137SeM (residues 67–157) the crystal packing was different from that of 2WJ7, even though crystallisation conditions were similar (Fig. S2). Both the higher-resolution L137SeM αB ACD structures are very similar to the wild-type αB ACD (2WJ7), with the RMSD of each construct (when residues 73–147 are overlaid) equal to 0.68 Å^2^. This shows the tolerance for mutation L137M, a substitution that changes the crystal packing, but not the core ACD structure. Therefore, the R120G mutation site was engineered into the longer L137M construct, but for simplicity, from now on the R120G/L137M structure will be referred to as R120G. The RMSD between the high-resolution 71–157 αB ACD (2 Å resolution) and the R120G ACD (2.5 Å resolution) is equal to 1.589 Å^2^.

**Fig. 2:**
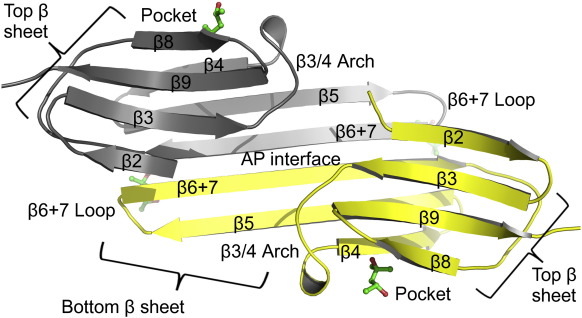
The structure of the α-crystallin domain of αB-crystallin R120G/L137M. The structure is annotated with the structural features of ACDs. The β-sheets are labeled and the hairpin loop connecting the β6 + 7 and β5 strands is indicated. One of the loops that link the two sheets in each monomer (strand β3 to β4) is shown as the β3/4 arch. The side pockets at the edges of β4 and β8 are labeled, and the AP dimer interface is labeled. The MPD molecules in the β4/β8 pockets and below the loops are depicted as green sticks.

The X-ray structure of the ACD from αB-crystallin R120G is very similar to that of the wild type, being composed of strands β2–β9.^40,41,44^ It is shown annotated with features common to ACDs in Fig. 2. There are highly ordered methyl pentane diol (MPD) molecules bound near the β6 + 7 loops and in the side pockets, where they mimic the binding of an isoleucine in the I-X-I C-terminal motif.^41^

There are now coordinates for several vertebrate sHsp ACD dimers in the Protein Data Bank (PDB),^40–42,44^ which show structural differences. When two wild-type human αB ACDs (2KLR and 2WJ7) are compared, there are significant differences between them: these structures were determined at different pH, with one being an excised ACD dimer solved by X-ray crystallography and the other an NMR structure solved in the context of a large oligomeric assembly. We will now compare all the vertebrate sHsp structures to help elucidate those changes caused by the R120G mutation. We begin by analysing discrete parts of the structure that show change and then attempt to build a more complete picture of how the ACD dimer performs its role.

### Variability of the β2 strand

**Fig. 3:**
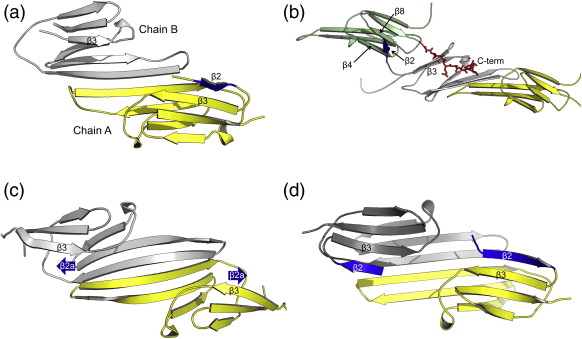
Variability and strand swapping at the β3:β2 position. (a) In human αB (2WJ7) the amino acids adjacent to β3 form the β2 strand in one chain of the dimer. (b) In rat Hsp20 (2WJ5) the β2 strand is not part of a β-sheet and (together with the construct N-terminus) is extended in both chains, allowing a hydrophobic side chain to bind in the β4/β8 pockets of an adjacent dimer. The C-terminal extension of a symmetry-related chain now binds to the β3 strand (shown as red sticks). (c) In human αB (2KLR) the intrachain β2 strands are not present; however, interactions from upstream N-terminal residues from another dimer are present. (d) The R120G structure showing intrachain β2 strands for each chain.

The variable sequence location and conformation of the β2 strand impacts on the accessibility of the β3 strand. In the R120G and the zebrafish αA (3N3E) crystal structures, the β2 strand is ordered and forms a curved β-sheet at a lattice interface, keeping the adjacent β3 strand covered (Fig. S3). In human wild-type αB-crystallin ACD (2WJ7), the β2 strand can be ordered or disordered (Fig. 3a). In the oligomeric structure of human αB solved by solid-state NMR (2KLR), β3 strand residues Asp78 and Val76 are paired with upstream residues Ser59, Trp60 and Phe61 (termed β2a) from the N-terminal extension of a separate dimer. The residues equivalent to the β2 strand form a connection between β2a and β3^44^ (Fig. 3c), referred to as heterogeneity region 1 (HR1), due to the many structural conformers that this sequence adopts.^44,48^

In the rat Hsp20 (HSPB6) structure (2WJ5), the sequence corresponding to the β2 strand is not hydrogen bonding with β3, instead it extends across to a partner domain in the lattice and inserts a hydrophobic side chain, topologically equivalent to Met68 in the β2 strand of human αB-crystallin, into a β4/β8 side pocket. The absence of both β2 strands from the two top sheets of rat Hsp20 ACD dimer creates space for the binding of the (C-terminal) peptide into the shared groove (Fig. 3b).

Taken together, these interchain interactions in the structures highlight the role in oligomerisation that the N-terminal and β2 strand sequences might play and the opportunity for β3 strand to bind variable partners.

### Variability of the bottom β-sheet

**Fig. 4:**
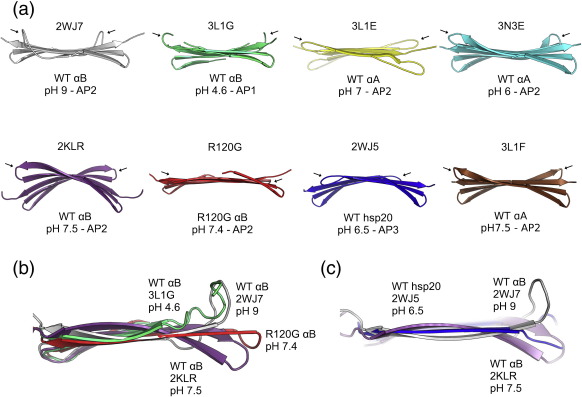
The bottom sheet, strands β6 + 7, β5 and β4, is variable. (a) The wild-type human αB ACD, 2WJ7 (grey); wild-type human αB ACD and C-terminal motif, 3L1G (green); wild-type bovine αA ACD and C-terminal motif plus zinc, 3L1E (yellow); wild-type zebrafish αA ACD and C-terminal motif, 3N3E (cyan); the solid-state NMR ACD structure from human αB full-length oligomers, 2KLR (purple); human αB ACD, R120G (red); wild-type rat Hsp20 ACD, 2WJ5 (blue); wild-type bovine αA ACD and C-terminal motif, 3L1F (brown). Note that some structures have a flat sheet, while other structures have varying degrees of curvature. The register is listed below the structures, as AP1, AP2 or AP3. The pH of the crystal is also noted below the sheet. The loop position is indicated with an arrow. (b) An overlay of the β6 + 7 and β5 strands from the αB structures, looking from the AP dimer interface showing the β6 + 7 loop position and the length of the β6 + 7 strands. The loop between the β6 + 7 sheet and the β5 strands sits up at 90° to the sheet in the wild type at pH 9 (grey) and pH 4.6 (green). The R120G (red) has longer β-sheets flanking the loop, and the loop does not sit up like those of the wild type. (c) The β6 + 7 strands from 2WJ7, 2WJ5 and 2KLR are overlaid. The 2KLR shows the marked curve in the strand, whereas the 2WJ7 and 2WJ5 have essentially flat strands. The curvature observed in rat Hsp20 [in (a)] is due to the register shift, and not intrinsic curvature of the β-sheet.

The length of the β6 + 7 strand is determined (in part) by the number of H-bonds to the β5 strand and is correlated with the length of the intervening β6 + 7 loop. In wild-type αB ACD (2WJ7), at pH 9 the loops sit up, at almost 90° to the β-sheet (Fig. 4a and b), whereas in the wild-type αB structure solved by solid-state NMR (2KLR) and in R120G, the shorter loops sit below the plane of the β-sheet. The changes in loop conformation change the surface structure of the domain around the AP interface.

The αB-crystallin ACD R120G dimer structure is in the same AP register as the pH 9 wild-type αB-crystallin ACD (2WJ7) and the pH 7.5 full-length αB-crystallin solid-state NMR structure (2KLR) and is close to physiological at pH 7.4. The AP interface is formed by β-sheet strands β6 + 7, β5 and β4 extending with the equivalent strands in the other chain into a larger β-sheet, referred to here as the bottom sheet. There is considerable flexibility in the structure of this extended sheet (Fig. 4a). It may be flat (3L1G, 3L1E and 2WJ7). It may be curved due to the register of the individual sheets changing, such as rat Hsp20 (2WJ5), where although the strands remain fairly flat (Fig. 4c), the two monomers have moved further away from the midpoint (into register AP3), creating a concave and convex surface (Fig 4A). Then, finally, the β-sheets may have an intrinsic twist, as seen in the solid-state NMR structure (2KLR) and, to a lesser extent, the two αA structures (3N3E and 3L1F) (Fig. 4a and c).

It has been suggested that the curvature of the β-sheet formed by β4, β5 and β6 + 7 is pH dependent^44^ based on the difference in curvature between the NMR structure at pH 7.5 (2KLR) and the crystal structure at pH 9 (2WJ7). However, the wild-type αB ACD crystal structures (that are now solved) are both flat at pH 9 and pH 4.6 (3L1G), and the αA structures at physiological pH are nearly flat (3L1E and 3L1F), making it unlikely that pH is solely responsible for the variability seen in the curvature of the bottom sheet (Fig. 4).

### The dynamic AP interface

The AP dimer formed about the β6 + 7 strands has been observed in three different registers in the vertebrate sHsp structures^41^ (Fig. S1), with the AP1 register having the greatest overlap and AP3 the least. In addition to the variation in the area of the AP interface due to the different registers, there is also the impact of the length of the β6 + 7 strand (Fig. 3b). The length of this strand is correlated with the number of H-bonds across the AP interface and therefore the dimer interface area. The longer β6 + 7 strands of the pH 7.5 structure (2KLR) and the R120G structure form a more extensive interface than the pH 9 structure (2WJ7). The surface areas of the various ACD dimer AP interfaces were analysed by PISA.^49^ In the wild-type αB-crystallin (67–157) structure (2WJ7), there are five chains in the asymmetric unit of the crystal C2 cell, forming three AP2 interfaces (two noncrystallographic and one crystallographic) with an average area of 633 Å^2^. In the solid-state NMR structure of the full-length αB-crystallin structure (2KLR), the ACD dimer interface (also in AP2) has an area of 895 Å^2^. With an interface area of 982 Å^2^, the R120G has a greater interface than any of the wild-type αB structures, even when the register and β6 + 7 sheet length are considered. This increased surface area is due to increased intermolecular interactions, described below. This increase in the dimer interface is also consistent with a tighter dimer interface for the R120G ACD dimer, shown by gel filtration at acidic pH.^40^

**Fig. 5:**
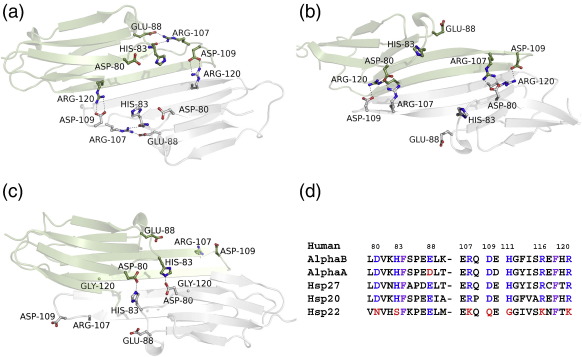
Conservation and variation of charged residues at the AP interface. (a) The wild-type αB-crystallin ACD (2WJ7) (pH 9). (b) The ACD dimer from full-length wild-type αB-crystallin (2KLR) (pH 7.5), determined by solid-state NMR. (c) The αB-crystallin ACD R120G structure (pH 7.4). The two chains are coloured light green and grey, and the residues forming key interactions at and around the interface are shown as sticks. Interactions are shown with dashed lines. (d) The truncated sequence alignment shows that in αA, αB, Hsp27 and Hsp20 the residues involved in the ion pairs at the AP interface are conserved, but vary in Hsp22. Human αB-crystallin numbering is shown above; residues coloured blue are those indicated on structures in (a)–(c), and residues that are not identical to the αB sequence are coloured red. The two conserved phenylalanine residues (84 and 118) are also shown in purple.

The AP dimer interface is likely sensitive to the environment, especially pH, as it is rich in physiologically titratable histidines. All the α-crystallin dimer structures (αA and αB) solved at physiological pH (7–7.5) are in AP2, as well as αB ACD at pH 9, whereas the more distantly related rat Hsp20 ACD at pH 6.5 (2WJ5) is in register AP3. Lowering the pH to 4.6 (3L1G), in the case of αB-crystallin, may cause the register to shift from AP2 to AP1, which results in disruption of the entire ion pair network, including the ion pairs between Arg120 and Asp109 (Fig. 5). In 2WJ7 and 2WJ5 these ion pairs are bidentate, whereas in 2KLR they are monodentate. The presence of bidentate ion pairs between Arg107 and Asp80 across the AP2 interface in 2KLR is a major structural difference between the two wild-type αB structures, the curved 2KLR and the flat 2WJ7, and may be responsible for the extensive curvature of the β6 + 7, β5 and β4 sheet of 2KLR (Fig. 5).^44^

While clearly this is a structurally dynamic interface, the two ion pairs between Arg120 and Asp109 across the dimer interface are conserved in the wild-type αB ACD at pH 9 (2WJ7) and the full-length solid-state NMR αB at pH 7.5 (2KLR) and rat Hsp20 (2WJ5) (Fig 1 and Table 1). This interaction is even maintained when there is a register shift, from AP2 to AP3 (rat Hsp20) and when there is an increase in pH. It is this pair of salt bridges that is disrupted in the pathogenic mutation R120G (Fig. 5).

Arg120 is part of an extensive ionic interaction network that crosses the dimer interface (Fig 5). Sequence alignments show a set of residues (Asp 80, His83, Arg107, Asp109, His111, Arg116, Arg120) that is conserved in αB, αA, Hsp27 and Hsp20, yet they are all different in Hsp22 (inset in Fig 5). This strengthens the case that these charged side chains are functionally connected and that Hsp22 has diverged. In muscle αB, Hsp27 and Hsp20 can form co-oligomers,^50^ and αA, αB and Hsp27 have been shown to co-oligomerise and exchange subunits *in vitro* in a temperature-dependent manner^51^ as have Hsp27 and Hsp20.^52^ Although co-assembly of Hsp22 with Hsp27 is less clear, there is cell biological evidence that mutant Hsp22 interacts more avidly with Hsp27 than wild-type Hsp22.^32^ If sHsps use this interface to bind unrelated protein partners (for insertion into networks) or nonnative protein substrates (as a chaperone), then the distinctive Hsp22 AP interface may facilitate binding of different partners and substrates from the other sHsps.

### Changes to the charged groove at the mutant AP interface

**Fig. 6:**
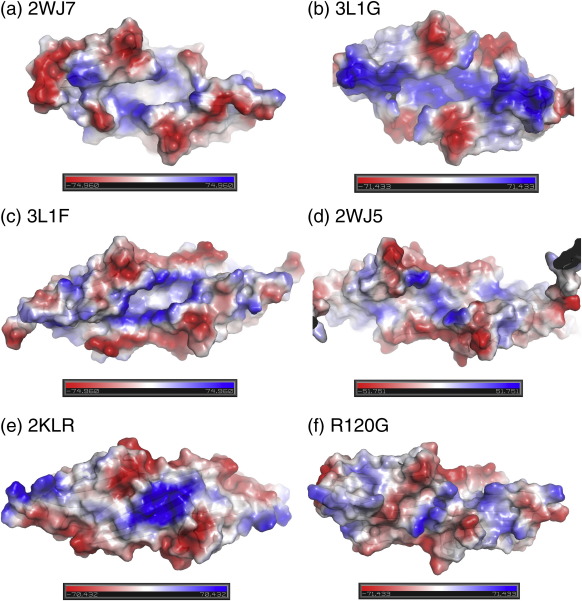
The conserved positively charged groove along the AP interface is lost in R120G. This groove is conserved in wild-type ACD structures of human αB [2WJ7 (a) and 3L1G (b)], bovine αA (3L1F) (c) and rat Hsp20 (2WJ5) (d). In the R120G, the groove is absent, due to ion pairs between Asp80 and His83, on the arches connecting top (β3, β9 and β8) and bottom sheets, β-sheets having moved closer to the centre in R120G. (e) The groove is closed in the ACD dimer from full-length wild-type αB-crystallin (2KLR).

**Fig. 7:**
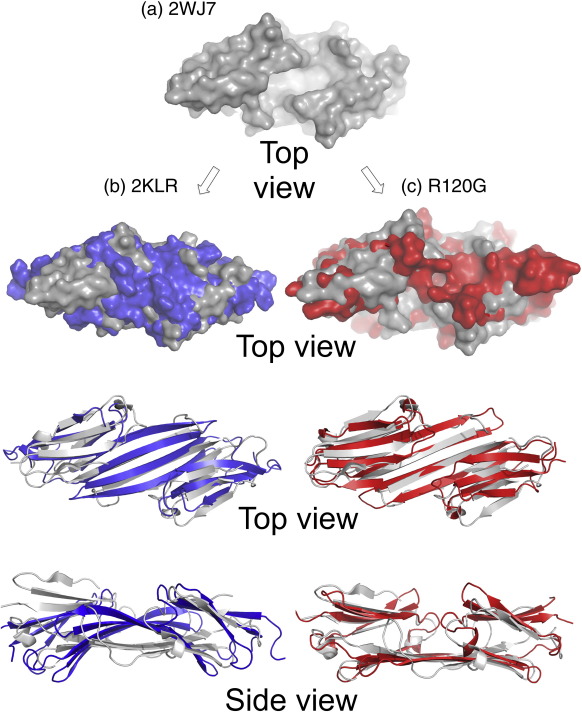
Closing the groove in two different ways. (a) The wild-type αB-crystallin ACD (grey) showing the deep groove. (b) In the full-length wild-type αB-crystallin (blue) the lower β-sheet has moved up into the space where the groove was in structure (a). (c) In the R120G αB-crystallin ACD (red) the β3–β4 arches have moved into the groove. In (b) and (c) the 2WJ7 structure is shown in grey, overlaid with the second structure shown in either blue (2KLR) or red (R120G).

The sHsp wild-type ACD X-ray structures all have a deep groove that runs down the AP dimer interface. The groove is maintained through sequence changes, register shifts and across a wide pH range (Fig. 6). The base of the groove is lined with four arginines, Arg120 and Arg116 on each of the β6 + 7 strands. The change in Arg120 to Gly120 results in the loss of two positive charges along the interface. This in turn results in loss of two interface ion pairs between Arg120 and Asp109 and gain of two new interface ion pairs between His83 and Asp80 (Figs. 5 and 6). These ion pairs between Asp80 and His83 are not observed in any of the currently available wild-type αB ACD structures. Both residues are on an arch between strands β3 and β4 that links the top sheet to the bottom sheet in the ACD on each side of the groove (Fig. 2). Movement of these arches toward each other to form two pairs of interchain salt bridges blocks the groove (Fig. 5). In the wild-type αB 2KLR structure the cleft is also absent, but in this case the bottom sheet curves up into the area between the top sheets (β3, β9 and β8) (Fig. 7).

The ability to open and close the groove may be facilitated by two phenylalanines that are conserved in all metazoan ACDs (purple in the alignment in Table 1 and the inset in Fig. 5). In the 3D structures, they are on the edge of the shared groove (αB residues 84 and 118) and interact by edge-to-face stacking of the side chains (Fig. S4). In the NMR structure of αB (2KLR), Phe118 also interacts with Arg116 across the AP interface *via* a pi–cation interaction.^44^ Arg116 makes an intrachain cation–aromatic interaction with His83 in αB (2WJ7) at pH 9, αA (3L1E) at pH 7 and αA (3L1F) at pH 7.5 (yellow in Table 1). This set of amino–aromatic interactions is embedded under the network of ionic interactions. As His83 is in a central position, it may respond to pH changes. In 2KLR, the interaction between His83 and Arg116 is broken, as it is in the crystal structures of rat Hsp20 (2WJ5) at pH 6.5, αB (3L1G) at pH 4.6 and αA (3N3E) at pH 6. In 2KLR, the altered position of Phe118 correlates with the bottom sheet being twisted (Fig. 7).

It is possible that the ACD dimer is able to fluctuate between open- and closed-groove conformations, with a closed state being favoured in the oligomer and the open state in the dissociated dimer. The structure of the R120G indicates that in the mutant this dynamism would be dampened.

## Discussion

Biophysical methods indicate that in solution αB-crystallin is polydisperse in size and in rapid dynamic equilibrium with subassembly species.^5,16,35^ We now propose, from analysing the 3D structures of ACD dimers currently available in the PDB, that the dimer itself exhibits conformational flexibility. Using N-terminally truncated sHsp sequences, crystallography has captured snapshots of dimers bearing a deep, shared groove at the dimer interface that is conserved in the structures of vertebrate ACDs of αB, αA and Hsp20.^40–42^ In the solid-state NMR structure, the αB ACD dimer has conformational differences from those solved by crystallography.^44^ The extended β-sheet, formed by strands β4, β5 and β6 + 7, is essentially flat in the crystal structures; however, in the NMR structure it is twisted, more like the shape of a hyperbolic paraboloid. The twisted bottom β-sheet curves up and fills the groove space. This form of αB-crystallin ACD dimer with the groove in a closed state either does not crystallise or exists only in the full-length highly oligomeric state. The crystal structure of R120G ACD dimer also reveals a closed groove, but in the mutant closure is caused by a pair of salt bridges between His83 and Asp80 close to the dimer 2-fold axis. The His83–Asp80 ion pair is not observed in any of the wild-type ACD dimer structures currently available. The two structures with closed grooves were solved at pH 7.4/5.

**Fig. 8:**
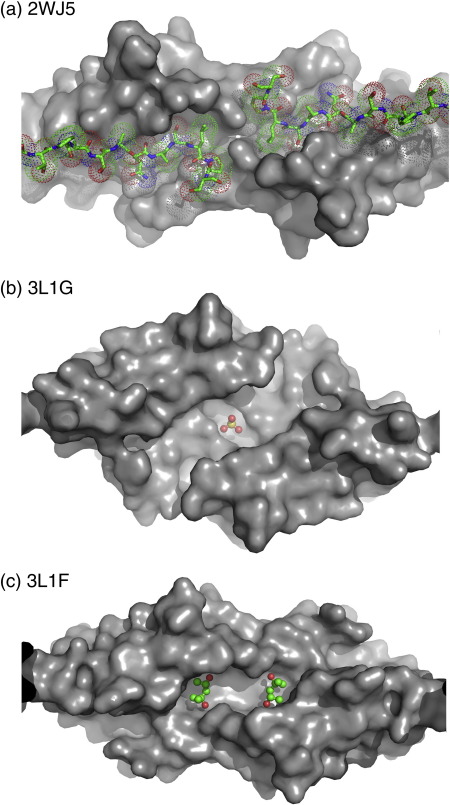
Groove as a binding site for other molecules. (a) The rat Hsp20 ACD has the C-terminal of a chain from an adjacent dimer bound in the groove. (b) The human wild-type αB ACD (3L1G) has a sulfate bound at the crystallographic 2-fold interface between the molecules. (c) The bovine wild-type αA has two MPD molecules bound in the groove.

In the structures of Hsp20, αA and αB, there are molecules (peptide, MPD and sulfate) that have co-crystallised in the AP groove (Fig. 8). This highlights the groove as a potential binding site for polypeptides and small molecules. The function of this dynamic groove down the AP interface may be to bind a phosphate-containing small molecule, which could allow regulation of these sHsps. In the wild-type αB structure (3L1G), a sulfate is bound at the 2-fold interface in the dimer (Fig. 8). Early experiments showed that ATP modulated the limited digestion of αB-crystallin with trypsin or chymotrypsin, which indicated that it could exert a conformational change of the ACD.^53^ When the residues with a nucleotide-dependent protease susceptibility are mapped onto the crystallin domain, they are located around the site of the sulfate molecule, lining the groove at the AP interface (Supplementary Fig. S5). It is possible that the sulfate in 3L1G may be bound in an ATP (or similar compound) binding site.

Recombinant full-length αB R120G mutant protein forms a soluble oligomer that is both larger and more polydisperse than wild type^33^ and is prone to aggregation and precipitation on handling.^34^ The mutation thus disturbs higher assembly of the oligomer, and the crystal structure of R120G ACD shows a dimer with a blocked groove. The N-terminal extension of αA has been shown to be necessary for its higher assembly.^51^ The solid-state NMR study provides evidence for the N-terminal extension playing a role in the oligomerisation of αB-crystallin by showing that the region around Trp60 (in what is termed the β2a strand) interacts with the β3 strand from a different dimer.^44^ It is possible that the N-terminal extension is involved in regulation of assembly. We speculate that these extensions may bind in the open groove in the full-length subassembly state, as the rat Hsp20 C-terminus binds in the Hsp20 groove in the crystal structure (in 2WJ5). In this scenario, the burial of the largely hydrophobic N-terminal extension may drive the dissociation of the dimer out of the higher oligomer.

How might these structural observations contribute to understanding how mammalian sHsps function? αB-Crystallin, in common with other more regular nonmetazoan sHsp assemblies, is in dynamic equilibrium in solution with subassembly species.^51,54,55^ The R120G with its new set of ion pairs would favour the ACD dimer in the closed form and disturb the subunit-exchange-driven assembly dynamics. Recently, an investigation of the quaternary dynamics of pea HSP18.1 has shown how heat can cause the regular assembly to increase in size and polydispersity, along with increased dissociation into subassembly species, in a reversible manner.^56^ These authors show evidence that this thermal plasticity correlates with chaperone activity. Stress in mammalian systems can be relayed by kinases, with serines in human sHsps often being phosphorylated. In Hsp27, there is clear evidence for their phosphorylation causing dissociation into smaller oligomers.^57,58^ In keeping with our speculative model of the released N-terminal extension binding the open groove of a dimer, the four arginines in the groove may bind with higher affinity to the phosphorylated N-terminus, favouring the dissociated state. The groove may also be a site of regulation, as reflected by a number of small molecules that have co-crystallised in it and from the evidence that ATP might bind here accompanied by a structural rearrangement.^53^ The depth of the cleft and its possible role in the function of wild-type αB-crystallin make this region a possible candidate site for drug design.

The R120G mutant is a defective chaperone and coaggregates with other destabilised proteins in chaperone assays.^33^ This may be due to the mutation disturbing the subunit exchange dynamics, although for αB-crystallin, there is evidence that the efficiency of exchange is only slightly decreased by the R120G mutation^59^ or is increased.^34^ The groove may also be a client substrate-binding site (chemical probes also label β3 strand sequence^45^) whose access is regulated through the dynamics of the assembly in ways that involve the dynamics of the N-terminal extension. In a well-characterised nonmetazoan sHsp system for detecting substrate-binding sites, both the N-terminal extension and β7 strand bound substrates.^60^ However, the solution state of the dissociated full-length αB-crystallin dimer is not yet accessible to standard structural methods, and the geometric locations of the groove and ACD pockets are not defined in the ambient or in the activated forms of αB-crystallin. Our provisional model for how sHsps might bind substrates involves unfolded polypeptide chains wrapping around ACD domains by docking into substrate-binding regions formed by the shared groove and β4/β8 pockets. Support for this model comes from the X-ray structure of an sHsp from a parasitic worm, where completely resolved N-terminal extensions wrap around a set of four ACDs.^61^

Forced expression of αB R120G is highly toxic to cells.^62^ It causes activation of the aggresome pathway,^63^ with formation of massively aggregated bodies in the cell, similar to the effects caused by blockage of the proteasome.^20^ Animal models that overexpress αB R120G in heart muscle cells have increased levels of amyloid and develop cardiomyopathy.^64^ The ability of R120G to form oligomers larger than wild type yet with some solubility may be the basis of its toxicity causing it to aggregate over time with crucial cellular components, possibly blocking or overwhelming the waste-disposal systems in muscle cells. Similar damage has been suggested to cause motor-neuron-specific neurite degeneration when the sequence equivalent mutation, Hsp22 K141N/E, is expressed in elongated cultured primary neuronal and glial cells.^65^ In the eye lens, where most cellular organelles have faded away, R120G likely causes cataract by binding to other crystallins, causing aggregation that then scatters visible light.

## Methods

### Protein expression and purification

Three protein constructs of human αB-crystallin that encoded the residues of the ACD were used: αB L137M (residues 71–157), L137M (residues 67–157) and R120G/L137M (residues 67–157). The mutations R120G and L137M were generated by QuikChange® PCR mutagenesis, both in the longer construct (residues 67–157). After purification and recombinant tobacco etch virus (rTEV) cleavage of the histidine (His) tag from this construct, all the recombinant proteins contained three extra residues at the N-terminus, GAM.

The L137SeM (71–157) and L137SeM (67–157) constructs in the pProEx™ Ht(b) vector were expressed in *Escherichia coli* grown in SelenoMet™ medium base (Athena Enzyme Systems) with a hexahistidine affinity tag and purified by Ni chromatography, rTEV cleavage, and then gel filtration, as previously described for the wild-type αB-crystallin domain.^40^

To purify the R120G/L137M protein, a slightly different purification was used. Cells from a 6-L culture were lysed in buffer A (0.5 M NaCl, 50 mM potassium phosphate, pH 8) containing 20 mM imidazole and an EDTA (ethylenediaminetetraacetic acid)-free protease inhibitor cocktail tablet (Roche) by a cell disruptor (EmulsiFlex-C5, AVESTIN), and then briefly sonicated to shear the DNA. The cellular debris and any remaining whole cells were removed by centrifugation (20,000***g*** for 30 min) and filtered through a 0.45 -μm filter. The protein solution was then loaded onto a preequilibrated 5 -mL HisTrap™ HP column (GE Healthcare). The unbound proteins were removed with 10 column volumes of buffer A containing 20 mM imidazole. The proteins were then eluted in a stepwise imidazole gradient of buffer A containing 100, 150  and 250 mM imidazole, with the recombinant protein eluting in the 250 mM fractions. These fractions were pooled and dialysed into buffer [25 mM Tris–HCl (pH 8), 100 mM NaCl, 1 mM DTT] at 4 °C. Then rTEV protease was added (100:1 ratio of substrate/protease) and incubated at 4 °C for 2 days to cleave the His tag. The protein was then dialysed into buffer A (also containing 20 mM imidazole) and the flow-through from a second preequilibrated 5 -mL HisTrap™ HP column was collected. This step removes the His tag, rTEV and any uncleaved recombinant protein that binds to the Ni column. The eluant was then further purified by gel filtration on a Superose75 HR10/60 column, in buffer (25 mM NaCl, 25 mM Tris–HCl, pH 8). Fractions containing the recombinant protein were pooled, concentrated to 16.5 mg/mL (as determined by *A*_280_ using an extinction coefficient of 1446 M^− 1^ cm^− 1^) and stored at − 80 °C.

### Crystallisation

Crystals were all produced using the vapour-diffusion technique; the conditions used for each construct are provided below. Following crystallisation, crystals were mounted in a loop in the drop solution (all conditions contained MPD concentrations sufficient for cryoprotection) and flash-frozen by immersion in liquid N_2_.

#### L137SeM αB (71–157) pH 9

One microlitre of protein at 20 mg/mL in buffer [25 mM Tris–HCl (pH 8.5), 200 mM NaCl] was mixed with 1 μL of mother liquor [40 mM bicine (pH 9.0), 40% MPD] and equilibrated against wells containing 50 μL of mother liquor and incubated at 4 °C.

#### L137SeM αB (67–157) pH 9

One microlitre of protein at 20 mg/mL in buffer [25 mM Tris–HCl (pH 8.5), 200 mM NaCl] was mixed with 110 mM bicine (pH 9.0) and 55% MPD and equilibrated against wells containing 50 μL of mother liquor at 16 °C.

#### R120G/L137M αB (67–157) pH 7.4

One microlitre of protein at 16.5 mg/mL in buffer [25 mM Tris–HCl (pH 7.5), 25 mM NaCl] was mixed with 1 μL of mother liquor (36% MPD, 0.18 M potassium chloride), giving a final pH of 7.4 in the drop solution. Sitting drops were then equilibrated against wells containing 50 μL of mother liquor at 16 °C.

### Structure determination

#### L137SeM αB (71–157)

Data were collected at the Diamond Light Source, beamline I02, on an ADSC CCD detector. Eight hundred images with an oscillation angle of 0.45° were collected at a wavelength of 0.9794 Å. Initial indexing indicated point group 422. Following integration with Mosflm^66^ and scaling with Scala,^67^ systematic absences showed the space group to be *P*4_1/3_22 with one molecule in the asymmetric unit (giving a Matthews coefficient of 2.0 and a solvent content of 38%). Data processing statistics are shown in Table 2.

The structure was solved by molecular replacement using Phaser^68^ and the wild-type αB-crystallin (PDB code 2WJ7) lacking the β2-strand as a model. The solution was in space group *P*4_3_22 and had a *Z* score of 19.6 and an initial *R*-factor of 45.6%. Rounds of refinement with Phenix,^69^ alternating with manual rebuilding with Coot,^70^ resulted in a final *R* and *R*_free_ of 20.4% and 22.1%, respectively. Refinement utilised automatic water picking, TLS refinement (with two domains corresponding to residues 10–58 and 59–87, or each of the two β-sheets) and refinement of the anomalous signal from the single ordered selenomethionine residue. An ordered MPD molecule was observed in the density proximal to Ser135 in the β4/β8 ACD binding pocket.

#### L137SeM αB (67–157)

Data were collected at the European Synchrotron Radiation Facility (ESRF), beamline 14-1, where diffraction for this crystal extended to 3.7 Å. Three hundred and sixty images with an oscillation angle of 1° were collected for each of three wavelengths (peak at 0.978 Å). Initial indexing showed the data to be in point group 222. Following integration with Mosflm and scaling with Scala, systematic absences indicated the most likely space groups to be *P*2_1_22 or *P*2_1_2_1_2. Six α-crystallin domains in the asymmetric unit of this cell result in a Matthew's coefficient of 2.59 and an estimated solvent content of 52%. Data collection statistics are provided in Table 2.

The structure was solved by molecular replacement using Phaser and the higher-resolution L137SeM (71–157) coordinates as a model. Initially, four chains could be placed with good density, with a *Z* score of 25.7 and an initial *R*-factor of 48.5% in space group *P*2_1_2_1_2. However, these four chains did not form a lattice, and inspection of the initial maps revealed areas of positive density between the layers of molecules placed by Phaser. An additional αB ACD dimer was placed in this density by inspection, thus completing the lattice. The L137SeM low-resolution data set, originally collected as part of a multiwavelength anomalous dispersion experiment, was intended for use in structure solution. A significant anomalous signal is present in these data that has been used as an independent source of experimental phases to confirm the location of the poorly ordered chains. There are small but significant (3.5σ) electron density peaks corresponding to SeMet 137 for both poorly ordered chains and also for SeMet 68 from molecule E in anomalous difference maps calculated using peak wavelength data and phases from the final refined model with the two weak chains omitted. In addition, there is weak but significant density to support the location of the poorly ordered chains in maps calculated using phases optimised with Phaser from data collected at the peak wavelength and the partial molecular replacement solution consisting of the four well-ordered chains. This density is strongest where the two additional chains make crystal contacts with the rest of the structure, as might be expected.

Refinement was carried out with Refmac 5.6^71^ utilising local NCS restraints and the new Jelly Body refinement protocol and was alternated with manual building using Coot. The final *R* and *R*_free_ were 21.3% and 27.7%, respectively. Density for possible MPD molecules was seen in the same location as for the high-resolution structure, close to Ser135 for the four ordered chains initially placed by Phaser. Additional density that could correspond to MPD molecules was seen on the other side of this serine for three of these molecules.

#### R120G/L137M (67–157)

Data were collected at ESRF, beamline 14.4, as 77 images, each with a 2° oscillation angle, at a wavelength of 0.87 Å. Data from 2.5  to 46.3 Å were integrated and indexed by Mosflm and then scaled with Scala (Table 2). The space group and point group were uncertain due to a combination of probable twinning and missing axial reflections. The apparent point group was *P*6, and the likely space groups were *P*3_*n*_ or *P*3_*n*_21, where the nature of the screw axis was not known. Solutions were determined by molecular replacement using the L137SeM structure (residues 71–157) in Phaser in a number of space groups. The solution in *P*3_2_21 refined to sensible *R*-factors. Building by hand in Coot and intensity-based twinning refinement in Refmac (final twin fraction of 47%) resulted in final *R* and *R*_free_ of 17.3% and 24.0%, respectively, with good geometry as determined my MolProbity.^72^ All structures were processed with the CCP4 program suite,^73^ and images were generated with PyMOL.

### PDB accession numbers

Coordinates and structure factors have been deposited in the PDB. For human αB R120G/L137M (67–157), the PDB code is 2Y1Z for the coordinate entry, and the code is R2Y1ZSF for the structure factors. For human αB L137SeM (67–157), the PDB code is 2Y22 for the coordinate entry, and the code is R2Y22SF for the structure factors. For human αB L137SeM (71–157), the PDB code is 2Y1Y for the coordinate entry, and the code is R2Y1YSF for the structure factors.

Figure S1 AP Dimer Interface Register Shift. (a) AP3 – wild-type hsp20 (2WJ5). (b) AP2 – wild-type αB (2WJ7) is shown, also in the AP2 register is 2KLR, 3L1E, 3L1F and 3N3E. (c) AP1 – wild-type αB (3L1G). Glu116 (or the equivalent residue in hsp20) is shown in yellow in all structures, to show the position of the monomers moving relative to each other. Arrow indicates the movement of the two monomers, relative to one another. When the dimer is in the AP2 register Glu116 is at the 2-fold axis of the dimer. The Cα atoms of αB Arg120 and Asp109 are 10.4 Å apart in 2WJ7, (pH9) and 10.1 Å apart in 2KLR, (pH7.5) allowing an ion pair to form between the two side chains. When the dimer is in the AP3 register in rat hsp20 (pH6.5), the sequence equivalent arginine to αB Arg116 is closest to the 2-fold axis, and the Cα atoms of the ion pair equivalent to αB Arg120 and Asp109 are 9.4 Å apart allowing an ion pair to form between the two side chains. When the dimer is in the AP1 register in αB ACD in 3L1G (pH4.6), Phe118 is closest to the 2-fold axis, the Cα atoms of Arg120 and Asp109 are 15 Å apart, a distance too large for an ion pair to form between the side chains. The dimer in AP1 register has the greatest degree of overlap.

Figure S2 Changing the Crystal Packing and Improving Diffraction with the Amino Acid Substitution L137SeM. (a) In the crystal asymmetric unit of wild-type human αB 67-157 (2WJ7), the five protein monomers A-E form long chains of dimers in the lattice, with few side contacts between these chains. (b) In the asymmetric unit of human αB 67-157 L137SeM, with six protein monomers A-F, the mutation L137SeM (in the β8 strand) has changed the crystal packing so that the new contacts between dimers form a more three-dimensional lattice arrangement than the wildtype αB ACD.

Figure S3 β2 Strand Packing in Crystals. (a) The β2 strand of chain B forms a crystal packing interface in R120G. (b) This same crystal packing is observed in chain A of the zebrafish αA ACD (3N3E).

Figure S4 The Conserved Phenylalanine Pair in ACDs. The phenylalanines are conserved in sequences of metazoan sHsp ACDs, and their stacking interaction within a domain is conserved in all the 3D structures. (a) Structural overlay of the ACD from human αB crystallins 2WJ7 (grey), 2KLR (blue), and bovine αB 3L1F (green) reveals a structurally conserved face-to-edge stacking between Phe118 in the β6+7 sheet and the Phe84 in the β3+4 arch. This interaction is maintained in the R120G structure (red). In 2KLR the pair is displaced in space due to the twisting of the bottom sheet. The paired phenylalanines are also viewed in three complete dimers in AP2 register and in the context of residues His83 and Arg116. (b) NMR structure of αB (2KLR). (c) R120G αB mutant. (d) Crystal structure of αB ACD (2WJ7).

Figure S5 Putative ATP Binding Site Mapped onto the wild-type αB ACD (3L1G). Residues that show a difference in accessibility for a protease (trypsin or chymotrypsin) when ATP is bound are shown as sticks. These cluster around the sulfate-binding site in the wild-type αB (3L1G).

## Figures and Tables

**Table 1:**
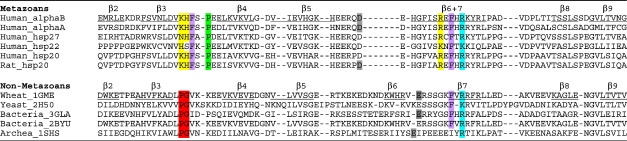
A structural and sequence alignment of the sHsp ACD

The β strands for the wild-type αB-crystallin (2WJ7) and wheat HSP16.9 (1GME) are underlined and labeled β2–β9. In nonmetazoans, there is a conserved ProGly (red) in the connection between strands β3 and β4 that packs close to the dimer 2-fold axis, leaving little room for bulky residues at these positions. In metazoans, this loop is in a different environment and the ProGly sequence is not conserved, with the equivalent residues in human αB-crystallin being Lys82His83 (yellow), with a conserved proline now located four residues later in the sequence (green). Also shown is the conserved arginine (cyan) in which mutations are associated with a number of diseases. Acidic residues that form an interchain ion pair with the conserved arginine are shown in grey. There are two structurally conserved phenylalanines that form a hinge-like interaction in metazoans along the edge of the groove (purple). The T-Coffee server was used to align the above sequences and their respective structures (PDB codes 2KLR, 2WJ7, 3L1E, 2WJ5, 1GME, 3GLA, 1SHS).^43^ The ion pairs are not indicated for 2H50 and 2BYU, as they are electron microscopy structures.

**Table 2:** X-ray data collection and refinement statistics

	L137M residues 71–157	L137M residues 67–157	R120G/L137M residues 67–157
*Crystal parameters*			
Space group	*P*4_3_22	*P*2_1_2_1_2	*P*3_2_21
Cell dimensions (Å)	*a* = 36.03, *b* = 36.03, *c* = 148.32	*a* = 67.12, *b* = 78.42, *c* = 130.67	*a* = 60.73, *b* = 60.73, *c* = 100.15
Angles (°)	α = β = γ = 90	α = β = γ = 90	α = β = 90°, γ = 120°

*Data collection*			
Wavelength (Å)	0.9794	0.978	0.8726
Resolution limit (Å)	2.0–35.01 (2.11–2.0)	3.7–67.24 (3.90–3.70)	2.50–46.56 (2.64–2.50)
Mosaicity	0.4	0.6	1.3
*R*_merge_	0.097 (0.322)	0.236 (0.66)	0.099 (0.536)
*R*_anom_	0.039 (0.066)	0.058 (0.169)	
Total no. of observations	184850 (27285)	100933 (14795)	69470 (9859)
Total no. unique	7272 (1018)	7861 (1109)	7582 (1087)
Mean *I*/σ*I*	25.6 (11.6)	8.7 (4.0)	19.4 (4.3)
Completeness	100.0 (100.0)	100.0 (100.0)	98.0 (97.8)
Anomalous completeness	100.0 (100.0)	100.0 (100.0)	
Multiplicity	25.4 (26.8)	12.8 (13.3)	9.2 (9.1)
Anomalous multiplicity	14.6 (14.7)	7.0 (7.1)	

*Refinement*			
Reflections	12582	7846	6777
Protein atoms in model	624	3342	1405
Solvent atoms in model	58	0	14
*R*_working_	0.204	0.213	0.173
*R*_free_	0.221^a^	0.277^b^	0.241^c^
RMSD from ideal geometry^d^			
Bond lengths (Å)	0.003	0.012	0.006
Bond angles (°)	0.67	0.28	0.98
Wilson *B*-factor (Å^2^)	21.4	56.1	43.7
*B*-factor of protein atoms (Å^2^)	33.6	88.8	31.2
*B*-factor of solvent atoms (Å^2^)	44.2		17.2
Ramachandran plot^d^			
Most favoured (%)	97.4	95.8	97.0
Outlier (%)	0.0	0.44^e^	0.6^f^
PDB code	2Y1Y	2Y22	2Y1Z

Overall values are listed, with the outer-shell values in parentheses. *R*_merge_ = ∑*|I*_*i*_ − *bI_i_N*|/∑*I*_*i*_. *R*_working_ = ∑|*F*_o_ − *F*_c_|/∑*F*_o_. *R*_free_ is the *R*-factor calculated for the cross-validated test set of reflections.

a *R*_free_ is 4.67% of reflections.

b *R*_free_ is 4.78% of reflections.

c *R*_free_ is 10.4% of reflections.

d As defined by MolProbity.

e Outliers are Glu67 in chain A and Glu106 in chain C, in poor density.

f Outlier is Asp109 in chain A, in poor density.
