# Supplementary material for: Crystal Structure of R120G Disease Mutant of Human αB-Crystallin Domain Dimer Shows Closure of a Groove
Source: J Mol Biol. 2011 Apr 22;408(1):118–34. doi: 10.1016/j.jmb.2011.02.020 (PMC3158665; doi:10.1016/j.jmb.2011.02.020)

**Supplementary Information**

**Figure Legends**

**Figure S1**. AP Dimer Interface Register Shift. **(a)** AP3 – wild-type hsp20 (2WJ5). **(b)** AP2 – wild-type αB (2WJ7) is shown, also in the AP2 register is 2KLR, 3L1E, 3L1F and 3N3E. **(c)** AP1 – wild-type αB (3L1G). Glu116 (or the equivalent residue in hsp20) is shown in yellow in all structures, to show the position of the monomers moving relative to each other. Arrow indicates the movement of the two monomers, relative to one another. When the dimer is in the AP2 register Glu116 is at the 2-fold axis of the dimer. The Cα atoms of αB Arg120 and Asp109 are 10.4 Å apart in 2WJ7, (pH9) and 10.1 Å apart in 2KLR, (pH7.5) allowing an ion pair to form between the two side chains. When the dimer is in the AP3 register in rat hsp20 (pH6.5), the sequence equivalent arginine to αB Arg116 is closest to the 2-fold axis, and the Cα atoms of the ion pair equivalent to αB Arg120 and Asp109 are 9.4 Å apart allowing an ion pair to form between the two side chains. When the dimer is in the AP1 register in αB ACD in 3L1G (pH4.6), Phe118 is closest to the 2-fold axis, the Cα atoms of Arg120 and Asp109 are 15 Å apart, a distance too large for an ion pair to form between the side chains. The dimer in AP1 register has the greatest degree of overlap.

**Figure S2**. Changing the Crystal Packing and Improving Diffraction with the Amino Acid Substitution L137SeM. **(a)** In the crystal asymmetric unit of wild-type human αB 67-157 (2WJ7), the five protein monomers A-E form long chains of dimers in the lattice, with few side contacts between these chains. **(b)** In the asymmetric unit of human αB 67-157 L137SeM, with six protein monomers A-F, the mutation L137SeM (in the β8 strand) has changed the crystal packing so that the new contacts between dimers form a more three-dimensional lattice arrangement than the wildtype αB ACD.

**Figure S3.** β2 Strand Packing in Crystals. **(a)** The β2 strand of chain B forms a crystal packing interface in R120G. **(b)** This same crystal packing is observed in chain A of the zebrafish αA ACD (3N3E).

# Figure S4. The Conserved Phenylalanine Pair in ACDs. The phenylalanines are conserved in sequences of metazoan sHsp ACDs, and their stacking interaction within a domain is conserved in all the 3D structures. (a) Structural overlay of the ACD from human αB crystallins 2WJ7 (grey), 2KLR (blue), and bovine αB 3L1F (green) reveals a structurally conserved face-to-edge stacking between Phe118 in the β6+7 sheet and the Phe84 in the β3+4 arch. This interaction is maintained in the R120G structure (red). In 2KLR the pair is displaced in space due to the twisting of the bottom sheet. The paired phenylalanines are also viewed in three complete dimers in AP2 register and in the context of residues His83 and Arg116. (b) NMR structure of αB (2KLR). (c) R120G αB mutant. (d) Crystal structure of αB ACD (2WJ7).

**Figure S5**. Putative ATP Binding Site Mapped onto the wild-type αB ACD (3L1G). Residues that show a difference in accessibility for a protease (trypsin or chymotrypsin) when ATP is bound are shown as sticks. These cluster around the sulfate-binding site in the wild-type αB (3L1G).

**Supplementary Figures**

Clark *et al*., Figure S1


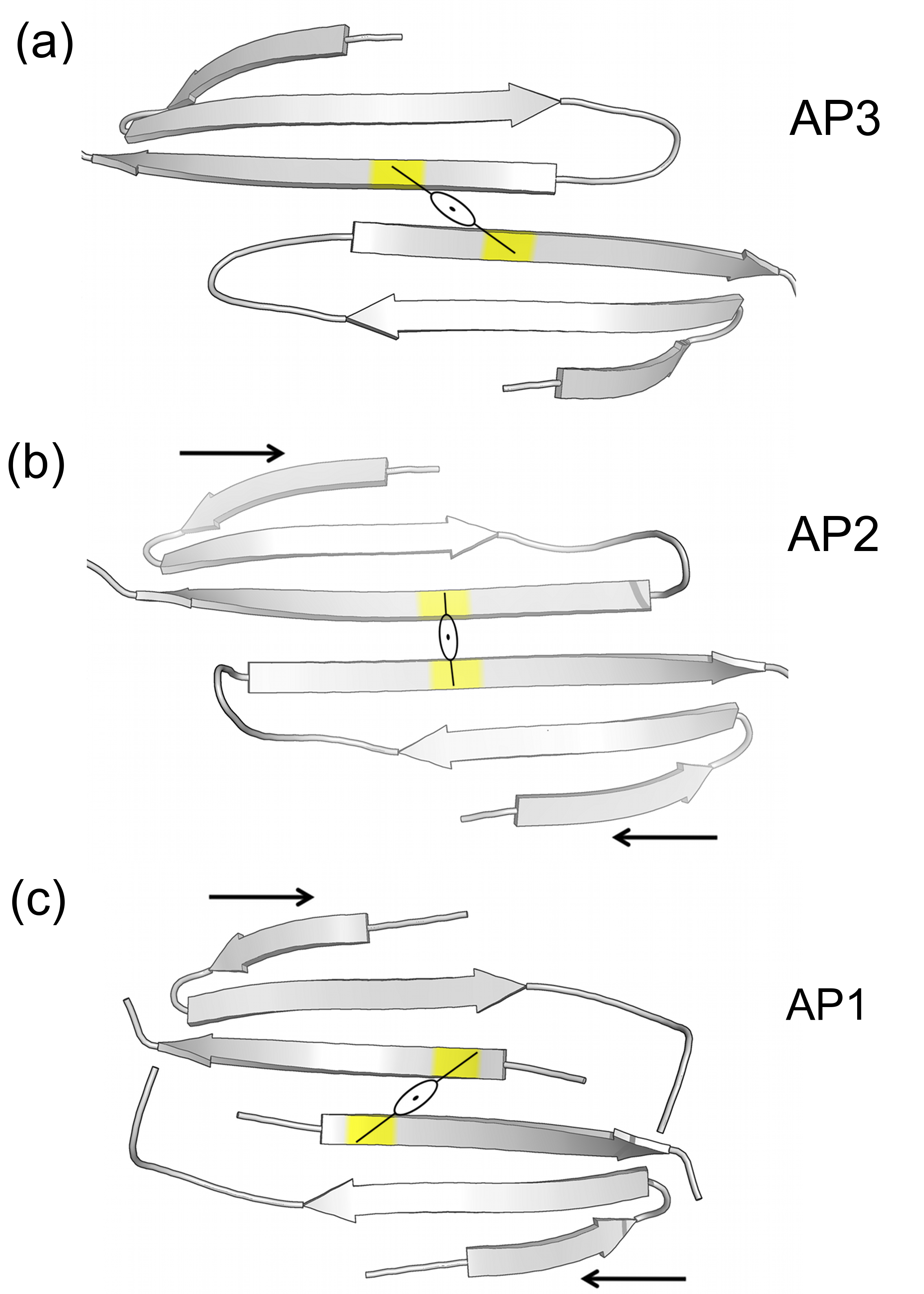


Clark *et al*., Figure S2

**
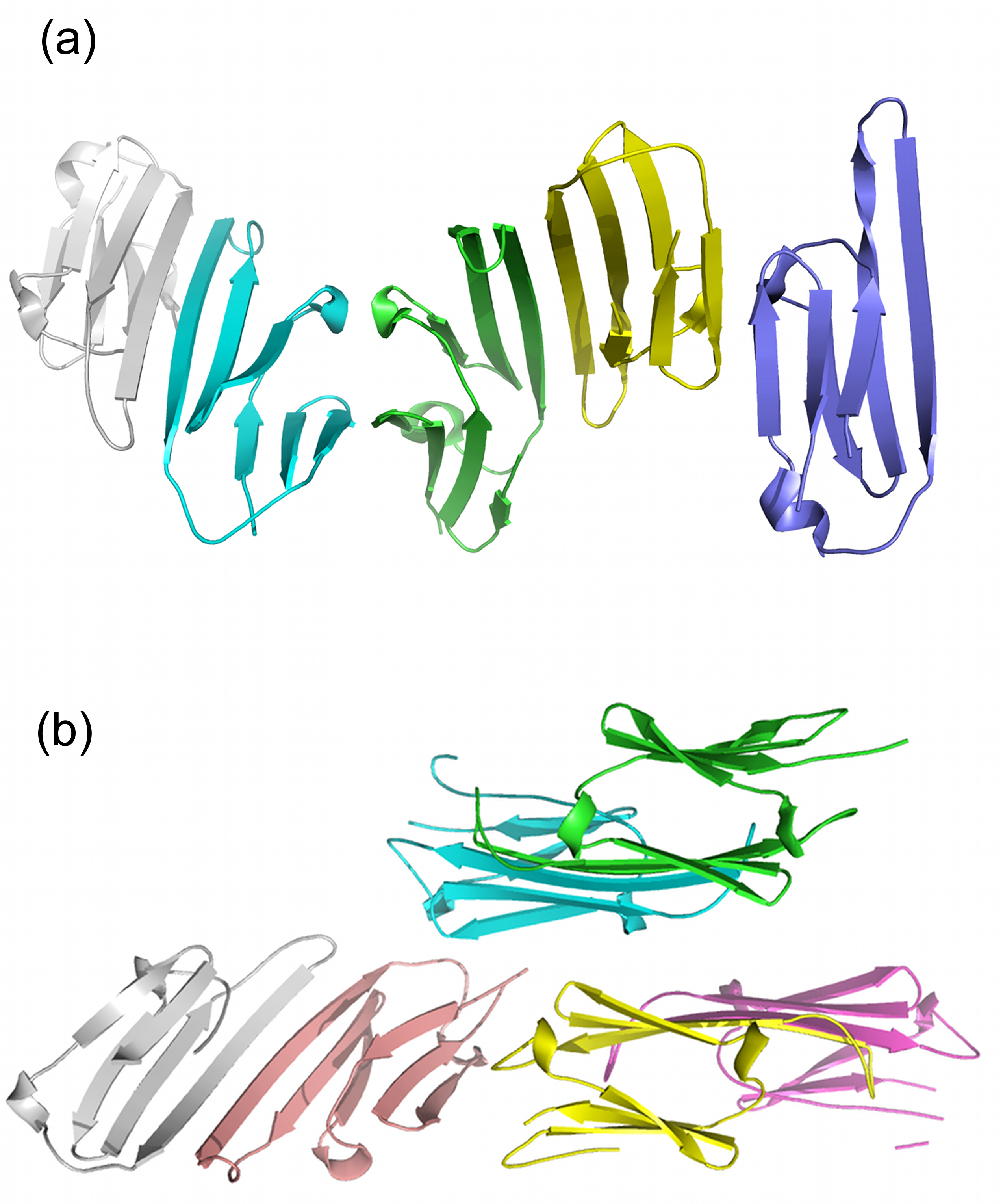
**

Clark *et al*., Figure S3


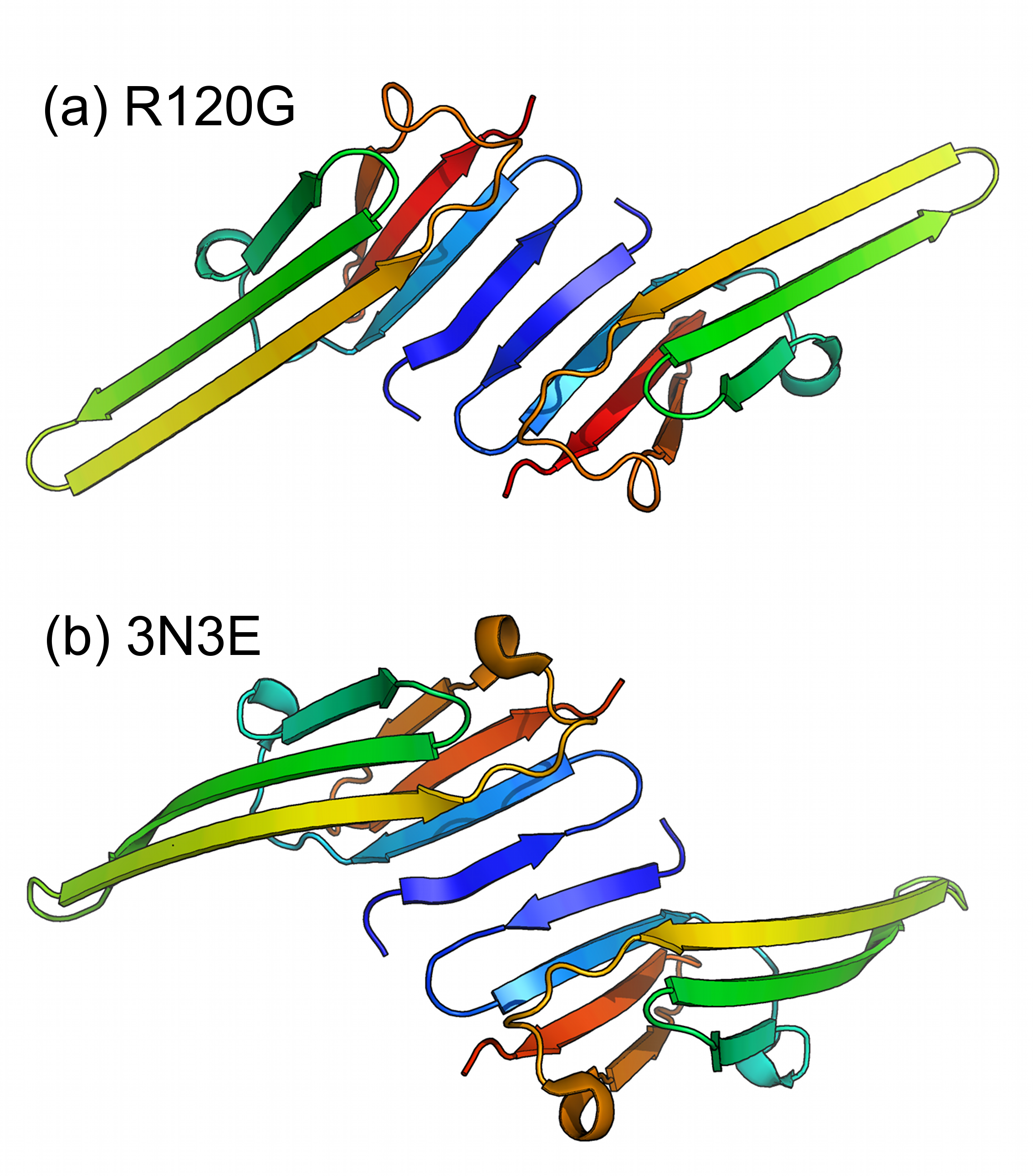


Clark *et al*., Figure S4


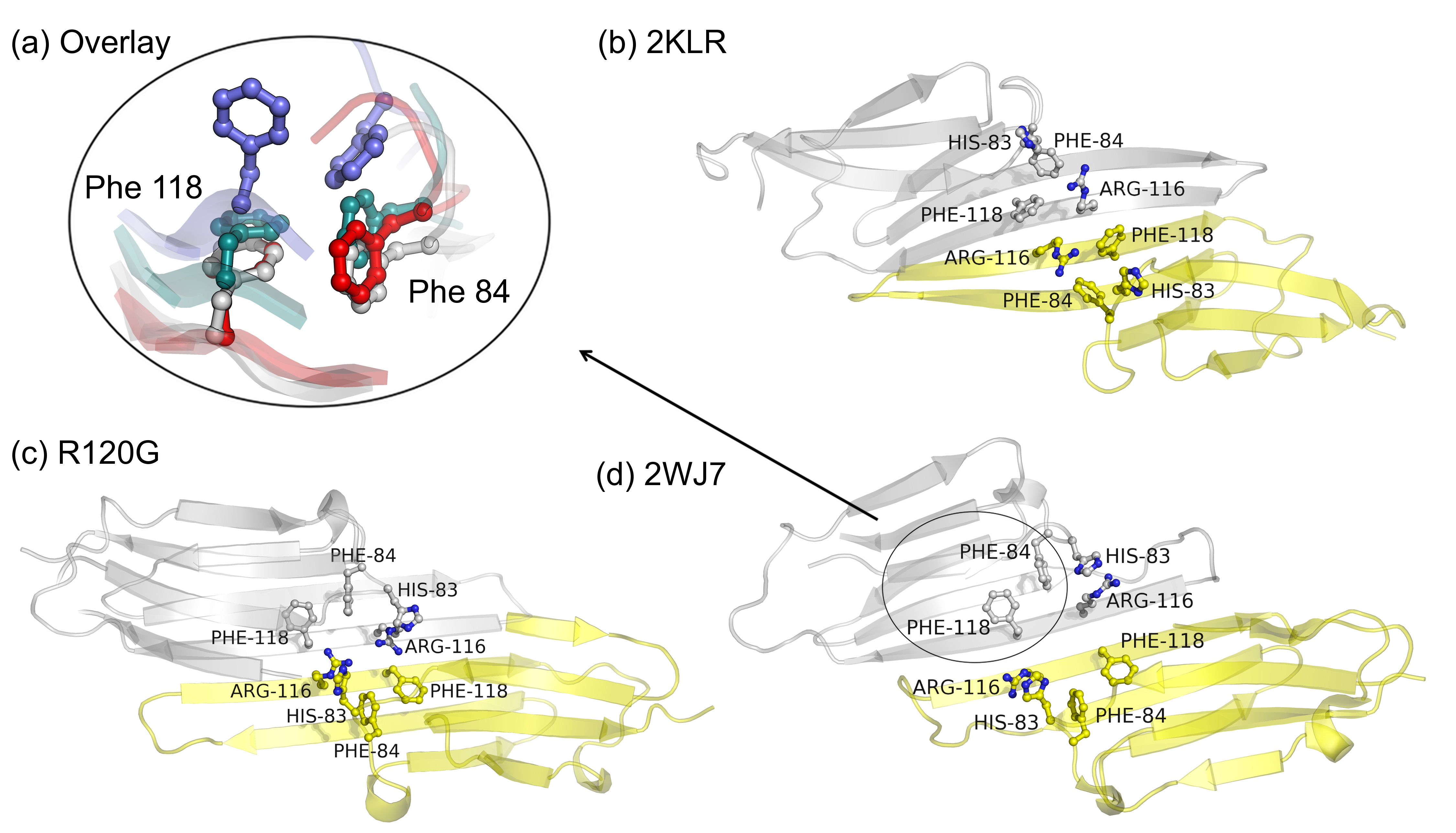


Clark *et al*., Figure S5


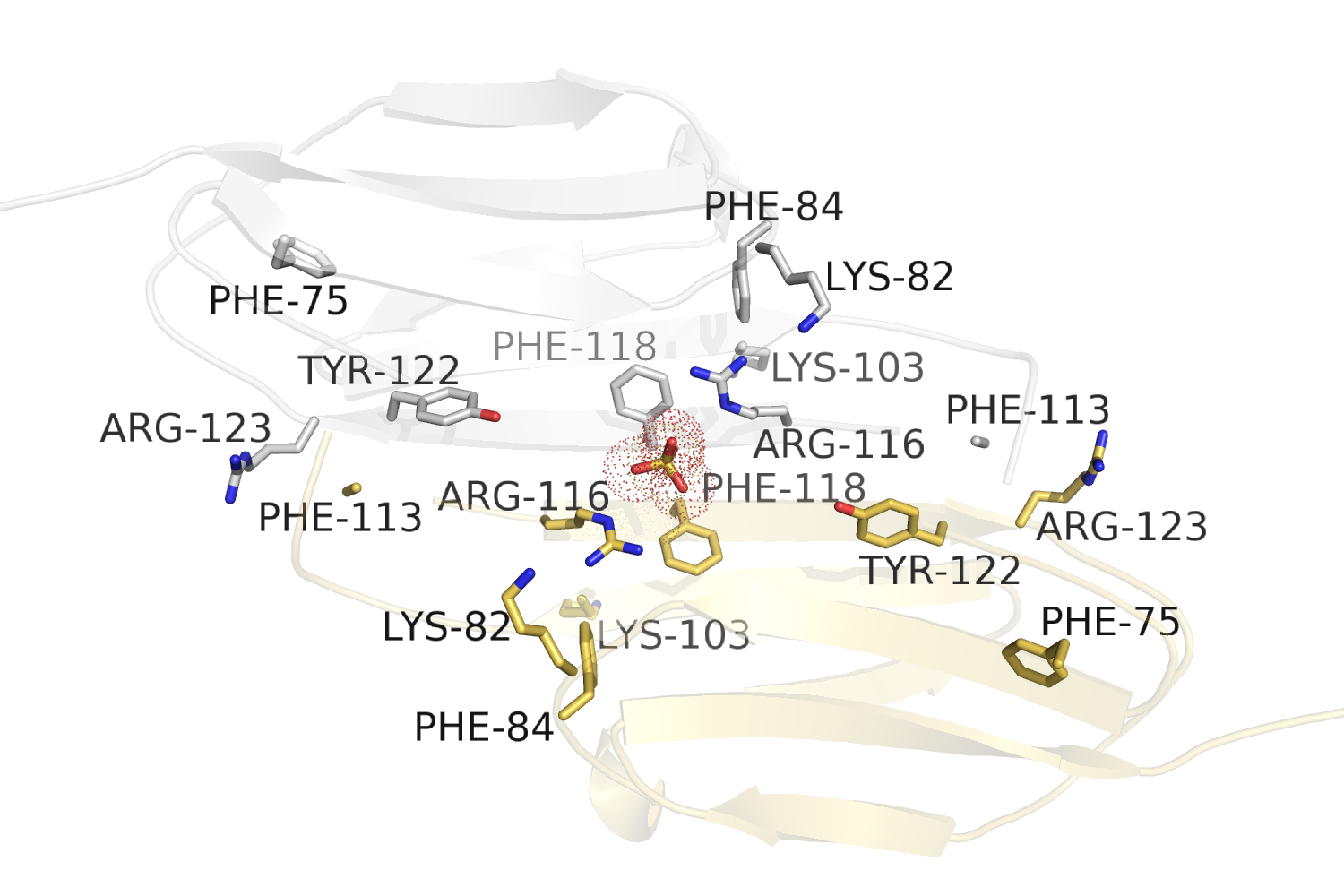

Supplement: Figure S1 — AP Dimer Interface Register Shift. (a) AP3 – wild-type hsp20 (2WJ5). (b) AP2 – wild-type αB (2WJ7) is shown, also in the AP2 register is 2KLR, 3L1E, 3L1F and 3N3E. (c) AP1 – wild-type αB (3L1G). Glu116 (or the equivalent residue in hsp20) is shown in yellow in all structures, to show the position of the monomers moving relative to each other. Arrow indicates the movement of the two monomers, relative to one another. When the dimer is in the AP2 register Glu116 is at the 2-fold axis of the dimer. The Cα atoms of αB Arg120 and Asp109 are 10.4 Å apart in 2WJ7, (pH9) and 10.1 Å apart in 2KLR, (pH7.5) allowing an ion pair to form between the two side chains. When the dimer is in the AP3 register in rat hsp20 (pH6.5), the sequence equivalent arginine to αB Arg116 is closest to the 2-fold axis, and the Cα atoms of the ion pair equivalent to αB Arg120 and Asp109 are 9.4 Å apart allowing an ion pair to form between the two side chains. When the dimer is in the AP1 register in αB ACD in 3L1G (pH4.6), Phe118 is closest to the 2-fold axis, the Cα atoms of Arg120 and Asp109 are 15 Å apart, a distance too large for an ion pair to form between the side chains. The dimer in AP1 register has the greatest degree of overlap. [file mmc1.doc]
